# Recapitulating the Cancer Microenvironment Using Bioprinting Technology for Precision Medicine

**DOI:** 10.3390/mi12091122

**Published:** 2021-09-17

**Authors:** Jisoo Kim, Jinah Jang, Dong-Woo Cho

**Affiliations:** 1 School of Interdisciplinary Bioscience and Bioengineering, Pohang University of Science and Technology (POSTECH), Pohang 37673, Korea; kjisoo@postech.ac.kr; 2 Department of Mechanical Engineering, Pohang University of Science and Technology (POSTECH), Pohang 37673, Korea; 3 Department of Creative IT Engineering, Pohang University of Science and Technology (POSTECH), Pohang 37673, Korea; 4 Institute for Convergence Research and Education in Advanced Technology, Yonsei University, Seoul 03722, Korea

**Keywords:** cancer biology, cancer model, tissue engineering, biofabrication, cancer microenvironment

## Abstract

The complex and heterogenous nature of cancer contributes to the development of cancer cell drug resistance. The construction of the cancer microenvironment, including the cell–cell interactions and extracellular matrix (ECM), plays a significant role in the development of drug resistance. Traditional animal models used in drug discovery studies have been associated with feasibility issues that limit the recapitulation of human functions; thus, in vitro models have been developed to reconstruct the human cancer system. However, conventional two-dimensional and three-dimensional (3D) in vitro cancer models are limited in their ability to emulate complex cancer microenvironments. Advances in technologies, including bioprinting and cancer microenvironment reconstruction, have demonstrated the potential to overcome some of the limitations of conventional models. This study reviews some representative bioprinted in vitro models used in cancer research, particularly fabrication strategies for modeling and consideration of essential factors needed for the reconstruction of the cancer microenvironment. In addition, we highlight recent studies that applied such models, including application in precision medicine using advanced bioprinting technologies to fabricate biomimetic cancer models. Furthermore, we discuss current challenges in 3D bioprinting and suggest possible strategies to construct in vitro models that better mimic the pathophysiology of the cancer microenvironment for application in clinical settings.

## 1. Introduction

Cancer is one of the leading causes of death globally, accounting for nearly 10 million deaths in 2020 [1]. Given the lack of a thorough understanding of cancer biology, considerable efforts have been devoted to the identification of cancer progression and therapy improvement. Many cancers, such as lung, colon, liver, stomach, and breast cancer, share common features and progression patterns, with respect to complex/heterogeneous microenvironments and increased tissue stiffness [2,3,4]. Cancer results from the transformation of normal cells into tumor cells, via genetic mutations, through a multistage process. Cells attain cancerous characteristics, such as induced angiogenetic proliferation, abnormal proliferation, and invasive and metastatic behaviors during the progression of the disease [5,6]. In addition, cancer cells living in a highly complex microenvironment are associated with surrounding stromal cells that they interact with, including endothelial cells, fibroblasts, inflammatory cells, and other extracellular matrix (ECM) components [7,8,9]. Moreover, cancer heterogeneity may affect the drug therapy provided to individual cancer patients, owing to the development of drug resistance from the subclonal genetic heterogeneity composition, leading to frequent therapeutic failure [10]. Therefore, it is necessary to comprehend the key factors surrounding cancer cells and the progression of cancer.

**Figure 1:**
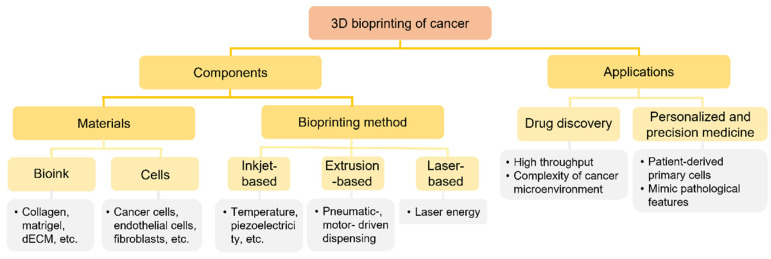
Tree diagram depicting the three-dimensional (3D) bioprinting process for cancer systems.

There is an urgent need for human cancer models that can recapitulate the complex cancer microenvironment to achieve the aforementioned goals. To address this challenge, animal models have been essential in cancer research [11,12]. However, animal models often fail because clinical trials do not reflect the genetic makeup of humans, owing to species-specific differences, or owing to their limited ability to emulate the complex physiology and progression of cancer in humans [12,13]. Therefore, animal models have been replaced by two-dimensional (2D) in vitro cancer models in cancer research. Although these models have contributed considerably to the accumulation of basic cancer biology knowledge, the proliferation, morphology, protein/gene expression, and drug responses associated with 2D and three-dimensional (3D) cancer models differ. Because cell–cell and cell–matrix interactions are important in cancer research, 3D models have more physiological in vivo relevance than 2D models, with respect to an in vitro approach to cancer biology [14]. Therefore, single and clumped cancer cells, such as spheroids, have been actively studied by coordinating several factors such as the microenvironment and mechanobiological system in a 3D environment. However, the interactions between cancer cells and surrounding stroma cells have not been considered sufficiently. For example, the lack of blood vessels in existing 3D models makes it impossible to supply nutrients considered important to bind cancer cells to a certain size and imitate in vivo studies. Therefore, several studies have used 3D bioprinting technologies to overcome these shortcomings. Current bioprinted in vitro human cancer models are considered as models because they can precisely mimic the complexity of the in vivo cancer microenvironment in humans by depositing suitable cells and the ECM, with respect to the composition of the cancer microenvironment (Figure 1). Numerous studies about the bioprinting technology used for the reconstruction of cancer systems have been published in recent decades. However, these have varied considerably in scope and in the adopted methods of classification and have not covered a comprehensive area. Therefore, this review discusses the essential elements of cancer system reconstruction with 3D bioprinting techniques, the components and technologies used to fabricate the cancer microenvironment, and the applications of the platforms developed. This review discusses some approaches that help create an appropriate cancer microenvironment and the latest technologies used to construct an in vitro cancer system, with emphasis on methods employing 3D bioprinting technologies, having the potential to construct complex tissue structures. Subsequently, the application of developed cancer models using engineered technologies is discussed.

## 2. Targeting Cancer Microenvironment for Reconstructing 3D Cancer Models

### 2.1. Biomaterials: Components for Modeling Cancer Microenvironments

**Figure 2:**
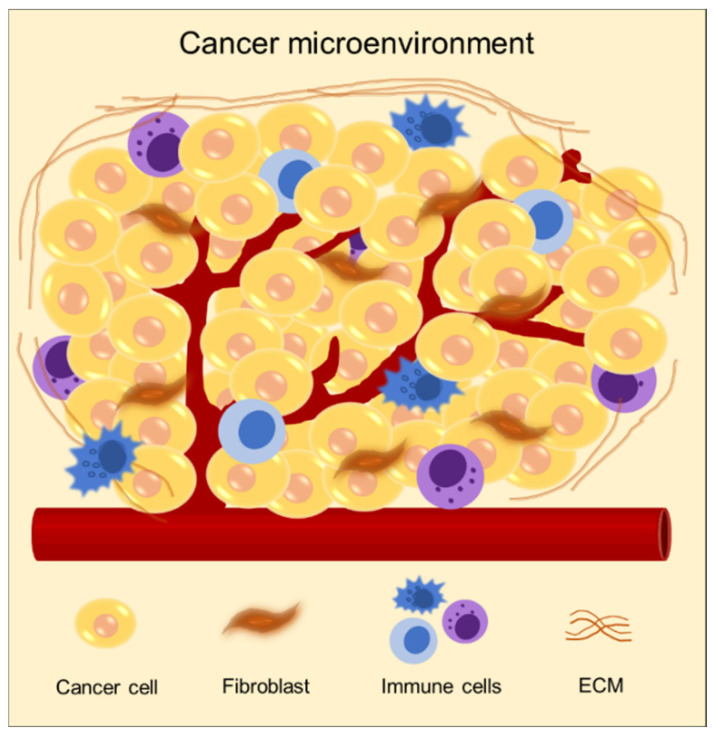
Schematic of characteristics of the cancer microenvironment composed of diverse cell types, including heterogeneous cancer cells, stromal cells such as fibroblast and endothelial cells, immune cells, and a dense extracellular matrix.

The cancer microenvironment comprises a complex ECM containing numerous components, including proteins, molecules, and cells (Figure 2) [15,16,17]. ECM provides biochemical and mechanical cues to cancer. It delivers biochemical signals to cancer cells to regulate gene expression and participates in determining the phenotype of the cell [18,19,20]. In addition, matrix stiffness plays a critical role in regulating cancer behaviors, such as cancer progression and metastasis through mechanical signaling [21,22,23]. Therefore, the selection of biomaterials in modeling cancer is required to attain an accurate recapitulation of the in vivo microenvironment of cancer.

#### 2.1.1. Collagen

Collagen is one of the most extensively used biomaterials. It constitutes a considerable proportion of proteins in the body and plays an important role in the formation of tissues and organs [24,25]. During cancer progression, there are many changes in collagen composition and orientation. Specifically, levels of collagen type I increase and those of collagen type IV decrease [26,27,28]. Therefore, collagen is used as a 3D cell-culture scaffold for bioengineering cancer models that emulate key microenvironmental conditions of in vivo cancer progression. Szot et al. developed a 3D, in vitro bioengineered cancer model in a prevascularized stage by culturing MDA-MB-231 human breast cancer cells in collagen type I [29]. Collagen type I facilitates invasive and uninhibited cell proliferation and formation of large cell clusters. Moreover, it promotes necrosis and hypoxia by modulating the collagen thickness to control oxygen and nutrient diffusion. Campbell et al. developed a 3D, collagen-based freeze-dried scaffold with axially aligned pores, to mimic the linearized collagen fibers in vivo [30]. This study demonstrated that MDA-MB-23 cells exhibit aggressive behavioral characteristics, including migration and invasion patterns, in anisotropic scaffolds, as compared with isotropic scaffolds.

#### 2.1.2. Matrigel

Matrigel, a hydrogel matrix comprising basement-membrane extracts obtained from Engelbret–Holm–Swarm mouse tumors, has been reported to promote tumor cell growth and has been used in several studies to model cancer systems [14,31]. Badea et al. demonstrated that spheroids of MDA-MB-231 cells have uniform morphologies, increased diameters, and good circularity characteristics, and they are associated with increased proliferation rates, in hypoxic conditions in Matrigel [32]. However, Matrigel containing ECM-specific chemical and biological components has not been completely investigated. Additionally, the lack of in vivo cancer matrix components, such as collagen type I or hyaluronan, is a challenge [14,33]. Some researchers have overcome these challenges by using the Matrigel–collagen hydrogel as a 3D in vitro culture system [34,35].

#### 2.1.3. Decellularized Matrix

Decellularized ECM (dECM) comprises several types of ECM molecules and preserves the original microenvironment [36,37,38]. In cancer, the composition of the ECM and the molecules bound to it differ as a function of tissue type and progression stage [39]. Therefore, studies have extensively focused on the development of in vitro cancer models that provide a tissue-specific environment to cancer cells. Tian et al. demonstrated the cancerous behavior of colorectal cancer cells, including their ability to form colonies spontaneously, and showed that they have molecular and phenotypic features similar to in vivo metastases using decellularized scaffolds that retained tissue-specific ECM components and bound signaling molecules [40]. Moreover, compared with the cells grown in and isolated from plastic, collagen, Matrigel, and lung BMSs, cells isolated from engineered liver metastases showed better liver metastasis formation patterns. This indicates that the biomatrix scaffolds recapitulate tissue-specific microenvironments and are more adapted to culture cells. In a similar study, breast cancer cells were cultured in human adipose tissue-derived ECM (hDAM) to investigate breast cancer growth and drug treatments [41]. Compared with the conventional cell culture methods, including 2D and 3D cultures in Matrigel, breast cancer cells exhibit prominent biomimetic behaviors in terms of cell growth, morphology, migration, and gene expression (such as CDH1, CDH2, vimentin), and drug resistance in the hDAM system. In addition, hDAM promotes the epithelial–mesenchymal transition (EMT), suggesting that hDAM mimics ECM in breast cancer in vivo and that breast cancer cells are surrounded by adipose tissue. IlKyoo et al. also revealed that decellularized patient-derived brain tissue (pdECM) can biomimic the glioblastoma multiforme (GBM) microenvironment [42]. In the pdECM-based 3D model, GBM cells show higher invasive patterns than collagen. Furthermore, invasion of GBM cells yielded considerably different morphological behaviors on materials on which cells a had more heterogeneous characteristic in pdECM than collagen. These studies demonstrated that decellularized matrices can closely mimic the in vivo tissue-specific microenvironment and are more suitable for cancer growth than traditional hydrogels, such as collagen I and Matrigel.

### 2.2. Engineering Mechanical Properties in Cancer Microenvironments

It has been reported that the cancer tissue microenvironments in the body are often stiffer than the normal tissue [43,44]. Mechanisms underlying cancer tissue stiffness have not been clearly identified, but as the stiffness surrounding the tumor microenvironment possesses important mechanical cues, several researchers have studied it over the past decade [45]. Baker et al. observed changes in cancer progression response in mammary epithelial cells (MECs) by modifying the collagen type I concentration [43]. Furthermore, the transformed cells yielded hyperproliferation and abnormal morphological responses with less defined boundaries. In a similar study, single-cell populations of MBA-MD231 breast cancer cells yielded differential rigidity responses in 3D matrices, and increased proliferation and metastatic behaviors were observed in stiffer matrices [46].

It was reported that overexpression of Yes-associated protein (YAP) and transcriptional coactivator with PDZ-binding motif (TAZ) were observed in cancer cells, and ECM stiffness regulated the activation of YAP and TAZ [47,48,49]. Jang et al. modulated the matrix stiffness range from 6.8 kPa to 0.5 kPa (approximately) of the storage modulus by digesting the alginate component with alginate lyase, while the remaining collagen composition demonstrated that the alteration of matrix stiffness could reverse the epigenetic changes [3]. The authors of this study found that YAP1 was upregulated in cancer tissues with stiffer matrices than in normal tissues. Moreover, matrix softening affects cell recovery and decreases expression of YAP, integrin β1, pFAK, and pMLC2, while recovering the methylation of YAP1. Aragona et al. used mechanical force to regulate the activities of YAP and TAZ [50]. YAP and TAZ were inhibited in the low-mechanical-stress region by F-actin capping and severing proteins manifested as losses of CapZ or Cofilin. The activities of YAP and TAZ were confirmed when cells were exposed to mechanical stresses, including stiff ECM matrices.

### 2.3. Reconstruction of Cancer Models with Heterogenous Cellular Populations

Cancer tissues are composed of multicellular organizations especially surrounding stroma, promoting the growth and invasion of cancer cells through various mechanisms [51]. The cancer stroma mainly comprises ECM components, fibroblasts, immune cells, and vasculature and is developed from native tissues via genetic alterations to provide a supportive environment for the cancer cell [52]. The importance of crosstalk between cancer and stromal cells, such as endothelial cells and cancer-associated fibroblasts (CAFs) was also reported [53,54]. Therefore, these factors should be considered in cancer models in vitro to emulate the in vivo microenvironment.

#### 2.3.1. Ability to Integrate Endothelial Cells

The critical role of endothelial cells in cancer progression has long been recognized. To sustain cancer growth, the stromal vasculature in cancer must support cancer cells with nutrients and oxygen. Cancer cells secrete proangiogenic factors, such as vascular endothelial growth factor (VEGF) and interleukin-8 (IL-8), and they induce angiogenesis and the formation of new blood vessels from the pre-existing vasculature [55,56]. The secreted angiogenic growth factors bind to the respective endothelial cell receptors and induce angiogenesis sprouting of endothelial cells, branching, and differentiation [55,57]. Moreover, it has been reported that cancer-induced new tumor blood vessels often alter the phenotype and gene expression and are often leaky compared with normal vessels [58]. Leaky vessels allow cancer cells to intravasate and metastasize easily to other parts of the body [59]. Therefore, focusing the angiogenetic pathways and sprouting of new vessels is vital for modeling cancer in vitro. Current engineered approaches, including microfluidic systems and bioprinting technology, have tremendous capability to develop well-organized engineered 3D tissues with endothelial and cancer cells. Pranay et al. developed a bottom-up engineered 3D vascularized cancer model using a polydimethylsiloxane (PDMS) microfluidic encapsulation device [60]. More than 2D cultured cancer cells, vascularized cancer which secreted VEGF had tube-like 3D vasculatures, and it assembled and formed around cancer by geometric guidance for the endothelial cells in their bottom-up cancer model. Agua et al. established the “tumor-on-a-chip” microfluidics platform that could grow cancer cells and endothelial cells to form vascularized microtumors [61]. On this platform, cancer cells were supported to form spheroids. Moreover, cancer cells in the vascularized model yielded similar metabolic heterogeneity results to those obtained in vivo.

Modeling a multiorgan system and the vasculature is a major challenge in the study of metastatic cancer [15]. Metastasis of breast cancer cells to bone was observed on a 3D printed vascularized cancer tissue model using a stereolithography printing system [62]. In in vivo models, breast cancer often metastasizes to bones [63], exhibiting vascularized bone and breast cancer cells printed on both sides with blood vessels located in the middle. Progression of transendothelial migration and the colony-forming behavior of metastatic breast cancer cells could be observed and monitored. Cell interactions among breast cancer cells, bone cells, and the vascular microenvironment were examined using this 3D printed multiorgan system.

#### 2.3.2. Ability to Integrate Fibroblasts

Fibroblasts are essential in many tissues for synthesizing ECM, producing structural frameworks, interacting with the epithelial cells, and secreting growth factors [64]. It was found that normal fibroblasts are activated and acquire a myofibroblast state with increasing expression of alpha smooth muscle actin (α-SMA) in wound healing states [53]. However, similar processes involving fibroblasts are activated during cancer and wound healing. These activated fibroblasts are named CAFs, and they actively participate in growth, invasion, and metastasis [65]. Several studies have attempted to reconstruct this unique environment of the cancer system with CAFs. Patient-paired sets of CAFs and normal fibroblasts (NFs) were cocultured with A549 lung cancer cells in an in vitro, 3D, coculture model to emulate the structure of in vivo cancer tissue and assess the functional effects of CAFs/NFs on lung cancer cells [66]. CAFs yielded higher α-SMA expression and increased levels of collagen gel contraction than NFs. Moreover, A549 lung cancer cells promoted to CAFs led to more effective collagen gel contraction, and CAFs enhanced the invasion of lung cancer cells in the collagen gel. Furthermore, Nair et al. demonstrated that CAFs support tumor maintenance and survival [67]. They established the sphere-forming cancer stem cells (CSCs), a key indicator shape of CSCs, and confirmed the tumorigenic potential of CSCs. They also demonstrated 3–7-fold increases in the CD133 expression levels. Furthermore, they induced the differentiation of CSCs and observed feeder-like myofibroblast cells surrounding the undifferentiated cell populations with an upregulation in expression of CAF markers, such as α-SMA and vimentin. Furthermore, it was observed that the treatment resistance of liver cancer cells was attributed to the effects of CAFs on the developed 3D organotypic coculture model [68]. An association between the increased expression of CAF markers in liver cancer and poor patient outcomes was also observed. Similar results were confirmed when a 3D in vitro model was used for coculturing of CAFs and when cancer cells exhibited increased drug resistance due to the induction of the CAF-derived soluble factors IL6, IL17A, IGF1, and IGF2 [68,69]. These results indicate that a 3D coculture model of CAFs and cancer cells can help understand the crosstalk between cancer cells and stroma.

## 3. Technical Approaches to 3D Cancer Model Construction In Vitro

### 3.1. Organoids as an Innovative Source of Discovery in Cancer Biology

Cancer organoid models offer a new direction in cancer research and have been actively studied in recent years. Organoids are 3D cultured multicellular clusters derived from stem cells to recapitulate the function of organs, such as multicellular differentiation behavior [70,71]. Remarkably, cancer organoids can preserve the genetic heterogeneity and phenotypes of the original cancer tissues, and they are being explored as suitable patient-specific models for drug screening. Currently, large collections of patient-derived cancer organoids have been developed to determine and predict the drug responses in patients. Thus far, 12 cancer organoids have been established [72,73]. In a recent study, Vlachogiannis et al. generated a large collection of cancer organoids to predict drug responses during clinical trials [74]. The authors of this study generated patient-derived metastatic gastrointestinal cancer organoids from 110 patients who were enrolled in phases I or II of clinical trials. Histopathological, molecular, and functional characterizations of patient-derived organoids were similar to original cancer cells. In a similar study, a biobank comprising a set of 20 colorectal carcinomas (CRCs) was established from patient-derived cells [75]. It was revealed that subclonal populations in organoid cultures were marinated compared with original cancer by cancer cell fractions. Moreover, a possibility of reducing the gap between cancer genetics and patient trials by showing that single organoids were sensitive to Wnt secretion inhibitors was suggested. These outcomes suggest that patient-derived organoids phenotypically and genetically resemble the cancer cells from which they were derived. Organoids could help in the decision-making process of clinical trials and promote personalized cancer treatment.

Although recent results in patient-derived organoid models are suitable, the coculture system has drawbacks. For example, additional cellular elements, such as the stroma, blood vessels, and immune cells, regarded as key factors of a cancer microenvironment system, are absent from the current organoid culture [73,76]. Organoids allow the development of a 3D cancer model with prolonged self-renewal and proliferation capacities. Thus, engineered approaches using organoids are required to develop a more biomimetic cancer model.

### 3.2. Microfluidic Modeling of the Cancer Microenvironment

**Figure 3:**
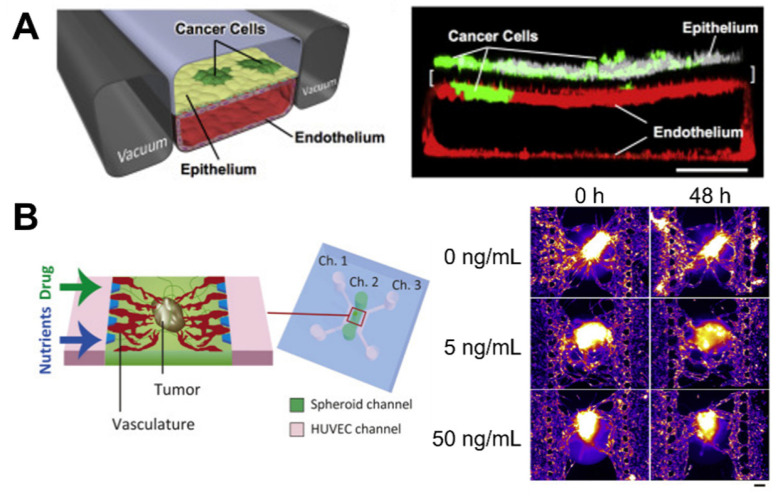
Microfluidic-based cancer-on-chip systems. (**A**) Lung cancer-on-a-chip system for the study of organ microenvironment-specific cancer behaviors, Reprinted with permission from ref. [82]. Copyright 2017 Elsevier. (**B**) Vascularized cancer-on-a-chip system for the evaluation of the effect of paclitaxel on cancer through the vasculature. Reprinted with permission from ref. [88]. Copyright 2020 Elsevier.

Microfluidic devices are cell culture devices containing perfused hollow microchannels for culturing living cells to recapitulate in vitro tissue- and organ-level structures and functions [77,78,79]. They generally comprise biocompatible and flexible polymers such as PDMS and are made using soft lithography to pattern microscale substrates. Controlling the fluid flow and patterning enables multicell cultures by delivering oxygen and nutrients. These advantages allow the fabrication of complex organs from specific tissue types, which are commonly referred to as organs-on-a-chip. One benefit of microfluidic devices is the fabrication of duct-like structures, such as blood vessels, with a continuous luminal flow that naturally occurs in vivo and is considered an important factor in the cancer microenvironment [80]. Recently, a complex organ-on-a-chip was designed, which contained endothelialized vascular channels and cancer cells that mediated tumor growth, expansion, angiogenesis, EMT, tumor cell invasion, and metastasis. Choi et al. studied the progression of an early-stage primary tumor within a normal epithelium using a breast cancer-on-a-chip system that provided an in vivo-like biochemical environment by allowing the flow of chemokines and cytokines through the lower channel [81]. In a similar study, organ-on-a-chip technology for human lung cancer emulated the growth and invasion patterns of non-small-cell lung cancer (NSCLC) by recapitulating the organ-specific microenvironment [82] (Figure 3A). Responses to epidermal growth factor receptor, MET protein kinase, and tyrosine kinase inhibitor therapy were observed in human patients during physically mimetic breathing motions, indicating high resistance to in vivo lung cancer therapy.

Some studies have focused on the development of cancer angiogenetic models. Angiogenesis is an important process during the growth and metastasis of malignant tumors [83]. Therefore, devices using organ-on-a-chip technology have been used to prompt endothelial cells to sprout in the hydrogel, to develop a perfusable angiogenic microvascular network [84,85,86], induce vascularized cancer to deliver drugs or nanoparticles, and predict the efficacy of the anticancer treatment [61,87,88,89]. For example, human umbilical vascular endothelial cells (HUVECs) were used to generate and sprout fibroblast and MCF-7 mixed spheroids seeded on a microfluidic device [88] (Figure 3B). Angiogenic sprouts were induced to construct a perfusable vascular network in a tumor spheroid that decreased the cell death in spheroids by supplying nutrients. Under perfusion conditions, the anticancer drug “paclitaxel” was administered, but there was no dose-dependent effect, in contrast to the result obtained in static conditions. This demonstrated the importance of flow in a vascular network for the drug screening platform. In addition, metastatic human breast cancer cells flowing through the perfusable microvascular networks simulated the extravasation process of cancer cells [90]. Cancer cells grown under hypoxic conditions exhibited higher extravasation rates than those grown under normoxic conditions (21% O_2_), thus indicating the role of hypoxia in promoting cancer cell extravasation during the operation of a microfluidic system.

Despite the advantageous characteristics of microfluidic devices, further improvements are needed. For example, limited alternatives are available in terms of materials to create such devices, and PDMS, which is most extensively applied in microfluidics, adsorbs hydrophobic molecules, leading to biased IC_50_ values in drug cytotoxicity tests [78,91]. Moreover, these devices are not used in clinical fields because of insufficient sensitivity, owing to small sample volume and sample purification processes [92,93].

### 3.3. Bioprinting: A Potential Technology for Fabrication of Biomimetic Cancer Models

**Figure 4:**
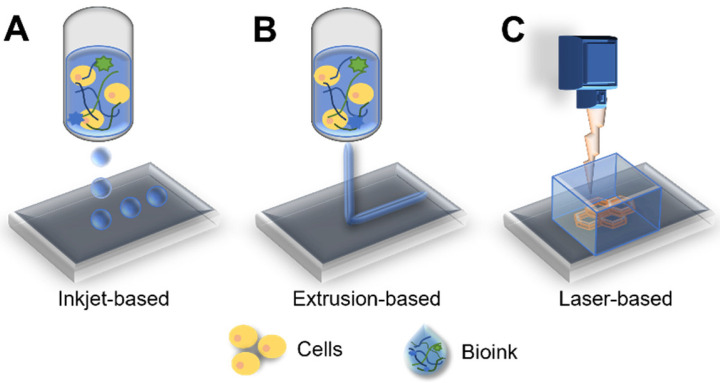
Schematics of printing approaches: (**A**) inkjet-, (**B**) extrusion-, and (**C**) laser-based bioprinting systems.

3D bioprinting is an additive manufacturing technology that deposits synthetic or natural biomaterials on a layer-by-layer basis using computer-aided design systems [94,95]. In the last decade, bioprinting technology has achieved remarkable advances. Bioprinting is mainly classified according to extrusion-, droplet-, and laser-based approaches, depending on the deposition method used (Figure 4) [96,97]. Extrusion-based bioprinting uses a pneumatic- or motor-driven dispensing system to deposit or laminate layered bioinks [97,98]. Droplet-based bioprinters are based on the deposition of droplets at different energy sources, such as temperature and piezoelectricity [99,100]. In the laser-based bioprinting approach, precisely patterned constructs are generated using laser energy [101]. All these bioprinting methods have an advantage in that they permit control over the cell distribution within the 3D structure [16]. Moreover, bioprinting has benefits, with respect to the modeling of coculture systems with other interstitial cell components in the cancer microenvironment. Wang et al. constructed a coculture model, including adipose-derived mesenchymal stem/stromal cells (ADMSCs) and human epidermal receptor 2 positive breast primary breast cancer cells (21PT), via the microextrusion bioprinting system, and the resulting coculture system was less sensitive to doxorubicin [102]. The ADMSC layer thickness was tuned to mimic in vivo obesity, and the apoptotic marker caspase-3 was lower in the group with thick layers than in the group with moderate layers.

**Figure 5:**
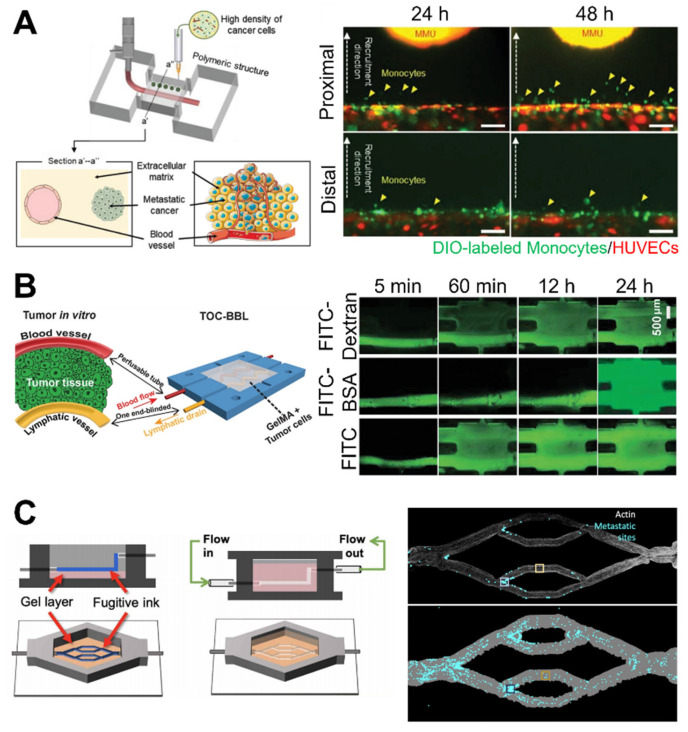
Examples of 3D bioprinted in vitro cancer models. (**A**) Bioprinted tissue-level cancer/vascular model used for the study of tumor metastasis based on precise-positioning. Reprinted with permission from ref. [111]. Copyright 2021 John Wiley and Sons. (**B**) Bioprinted tumor-on-a-chip system with blood and lymphatic vessel pair for the study of diffusion profiles of biomolecules and anticancer drugs. Reprinted with permission from ref. [112]. Copyright 2019 John Wiley and Sons. (**C**) Bioprinted vasculature for the study of the metastatic behavior of cancer cells. Reprinted with permission from ref. [113]. Copyright 2020 The Authors, some rights reserved; exclusive licensee American Association for the Advancement of Science.

Recent innovations in bioprinting technology include various approaches such as grafting microfluidic technology and automated high throughput of simplified or complex in vitro cancer modeling [103]. Using a microfluidic system, 3D printed perfusable vasculature could be fabricated using a sacrificial layer or coaxial printing system to emulate the dynamic cellular microenvironment by delivering oxygen, nutrients, and nanoparticles [104,105]. Fabricating a vasculature cancer model using the bioprinting technique involves printing the sacrificial layer for the vasculature, which is among the most extensively used methods [16,106,107]. This sacrificial structure is printed and deposited on the desired vascular pattern inside the hydrogel matrix or casting hydrogel around the sacrificial layer, followed by the removal of the remaining surrounding hydrogel matrix [108,109]. Campost et al. fabricated a vessel-like structure using a sacrificial layer cocultured with cancer spheroids [110]. Cancer spheroids led to continuous growth and promoted survival, and endothelization was observed in the bioprinted model. Hollow and perfusable channels were then prepared. Finally, the endothelial cells were seeded to mimic the endothelialized vasculature. In contrast, Kim et al. generated a cancer/vascular model with the use of an in situ and coaxial printing system [111] (Figure 5A). They fabricated the vasculature part using a 3D coaxial printing system with a 0.5% decellularized skin (SdECM) bioink. In situ printing technology was used to deposit the cancer cells at a desired distance from the printed vessel. Interactions between cancer and the vasculature were defined using position-controllable printing technology that lowered the distance structure to enhance the migratory and invasive properties. Furthermore, increased expression of representative matrix metalloproteinase (MMP) markers, including MMP2 and MMP9, was observed when the distance between cancer cells and the vascular bed was reduced. These findings demonstrate that interactions between cancer cells and the vasculature can modulate the behavior of cancer cells. Furthermore, coaxial printing systems can fabricate a tumor-on-a-chip with a hollow blood vessel and a lymphatic vessel pair [112] (Figure 5B). It is intended to fabricate perfusable blood vessels by opening both ends of the structure, while blocking one end of the lymphatic vessel, to recapitulate in vivo construction and function. In addition, this system yields the diffusion profiles of biomolecules and anticancer drugs with various degrees of accuracy.

Moreover, 3D bioprinted systems can explain the tumor cell behavior observed in cancer biology studies. Hynes et al. bioprinted endothelialized vascular beds to examine the metastatic behavior of circulating tumor cells (CTCs) [113] (Figure 5C). The bioprinting system was able to control the manufacture of complex geometries of vasculature. This is a key aspect in recapitulating the in vivo CTC environment. They demonstrated that CTCs were affected by the pressure and flow rate of the vessel, using computational modeling and a bioprinted analog model. The CTC attachment was greater on the arterial or “inflow” side of the device compared with the venous or “outflow” side.

## 4. Applications of Bioprinted Cancer Models

### 4.1. Cancer Models for Drug Discovery

The process for the discovery of new anticancer drugs is very inefficient. Although notable advances have been made in developing new anticancer drugs, approximately 5% of agents show sufficient clinical activity in phase III trials and can be approved for clinical usage [114]. The reasons for failure are inadequate preclinical models and a lack of understanding of the complex and heterogenous cancer system that leads to drug resistance [115]. The classic method used to identify the toxicity or activity of anticancer drugs is the use of a cell line or an in vivo model. However, cell lines are too sensitive to drug treatment and cannot predict drug resistance effects, and in vivo models are genetically different and cannot accurately recapitulate the cancer system in humans [11,79,116,117]. Recent approaches have been focused on the extraction of the genomic information via DNA sequencing for the development of precision medications for defined genetic aberrations; however, sequence results are commonly derived from untranslated regions, and there is currently a lack of relevant databases [118].

Therefore, the use of in vitro 3D human cancer models is regarded as an adequate route for drug discovery and precision medicine, as such models can precisely emulate the complexity of the in vivo cancer microenvironment in humans using suitable cells and ECM, in addition to other approaches. Bioprinting technology has enabled high-throughput, in situ, rapid fabrication of 3D, continuous optical printing in multiwell plates at the microscale [119,120]. Using direct in-well bioprinting and culturing of biomimetic human hepatocellular carcinoma scaffolds for high-throughput scale drug screening [119], Hwang et al. elucidated that the dose- and time-dependent doxorubicin toxicity of the fabricated scaffolds yielded results similar to those of previous studies. Moreover, other biomimetic models, including those for the vasculature, have been introduced to discover adequate drugs. Meng et al. developed a 3D, in vitro metastatic model with a functional vasculature and stromal elements [121]. The effect of the anticancer drug immunotoxin on this 3D metastatic model was compared with that on a 2D culture model using A549 cancer cells. Results revealed that the resistance to immunotoxin was higher in the developed model than in the 2D model. The 3D model with fibroblasts showed stronger resistance than other models, as drug molecules potentially bind to fibroblasts. These results indicate that a 3D model can provide more in vivo mimetic elements, including interactions between cancer cells and the surrounding stoma. These complex cancer models provide a more precise emulation of interaction with the drug and the consequent effects by providing a biomimic design with a high-throughput system; this facilitates the simulation of drug combinatorics studies and the measurement of effectiveness of the suppression of multiple pathways.

### 4.2. Personalized Cancer Models for Precision Medicine

Numerous studies demonstrated that high levels of intratumor heterogeneity and complexity lead to different cancerous behaviors in patients, even within the same cancer subtype, thus requiring different cancer therapy approaches [122,123,124,125]. Hence, a patient-specific model using patient-derived primary cells for precise medication has been studied. Patient-derived tumor organoids act as personalized cancer models that phenotypically and genomically resemble the cancer cells from which they were derived, and that enable high-throughput drug tests [126]. Studies on cancer organoids have established patient-specific cancer models of colorectal, gastrointestinal, pancreatic, prostate, breast, liver, glioblastoma, and lung cancer [74,75,127,128,129,130,131,132,133,134,135]. Patient-derived organoids can help determine appropriate treatments for individual patients, based on drug resistance test profiling, with respect to genetic changes in the individual. To examine the drug response of organoids during preclinical testing, Kim et al. established a living biobank of 80 patient-derived lung cancer organoids, with the five most common subtypes of lung cancer, for the recapitulation of histological and genetic features based on the evaluation of the initial and post-cytological quality [127]. They revealed that the drugs targeted specific genetic/molecular alterations and demonstrated synthetic lethality to pathogenic alterations via downstream signaling molecules. Moreover, patient-derived models of organoids of metastatic gastrointestinal cancers have been generated to compare the response of anticancer drugs with data from clinical trials of patients, as described above [74]. Using the generated organoids, it was found that the response of targeted agents or chemotherapy had 100% sensitivity, 93% specificity, 88% positive predictive value, and 100% negative predictive value. These results support the feasibility of using personalized medicine prediction approaches with established organoids to recapitulate the molecular profiles in patients. However, as organoid cultures lack coculture with the stroma, advanced engineered systems have been explored to construct cancer models, including other components of the cancer system.

Bioprinting technology allows the production and recapitulation of a highly biomimetic cancer system to identify patient-specific drug candidates [136,137]. Yi et al. developed a cell printing-based, patient-specific glioblastoma-on-a-chip [137]. The 3D printing system allowed compartmentalization of cancer and stroma to coculture the patient-derived glioblastoma cancer cells and HUVECs. Moreover, this model could emulate pathological features, including radial oxygen gradient, and the structural, biochemical, and biophysical properties of native cancer. In addition, radiotherapeutic and chemotherapeutic responses in 3D printed models correspond with those observed in patients, thereby providing evidence for the possibility of developing personalized cancer treatment research tools. This model exhibited significant differences in the resulting resistance levels and responses to concurrent chemoradiation with temozolomide, which corresponded to the clinical setting [137]. Recently, a scaffold-free tumor tissue model comprising multiple cell types was developed using 3D bioprinting to study tumor–stromal interactions using patient-derived pancreatic cancer cells, fibroblasts, and endothelial cells. These stromal cells improved the cancer maturation and self-organization, including vascular networks and cancer phenotypes such as ECM deposition. Upon assessing therapeutic responses to targeted PI3K/mTOR inhibitor BEZ235 in developed heterotypic cancer tissues, both stromal and cancer cells exhibited a decrease in pS6 following the treatment. These results suggest that engineered patient-specific cancer tissues with stromal cells, constructed using bioprinting technologies, can be used to study personalized treatment strategies based on drug response and cancer properties.

## 5. Conclusions and Future Prospects

The current review provided a representative overview of advanced in vitro model systems, in addition to bioprinting technologies, for application in cancer research. Advances in bioprinting technologies have helped establish in vitro 3D cancer tissue models by facilitating control over the biological properties of the models, including tissue construction and ECM composition, and they have provided improved alternatives to conventional drug screening methods, such as 2D cultures and animal models. Furthermore, contemporary bioprinting strategies have exhibited remarkable progress in personalized precision medicine endeavors by facilitating the fabrication of complex cancer systems.

Although current cancer modeling strategies have demonstrated tremendous progress, many challenges remain, with respect to the modeling of completely biomimetic cancer models that recapitulate the extremely complex and heterogeneous cancer microenvironment and the organ level of drug toxicity. Therefore, future research should focus on the fabrication and reconstruction of improved multifunctional cancer systems via incorporation of more realistic stroma to activate cancerous behaviors. Moreover, even though continuous improvements in bioprinting technology have demonstrated it to be a valuable tool for clinical applications, advancements are still needed to recapitulate pathological features at the patient level, to improve the accuracy of simulation of drug reactions.

However, as the technology underlying bioprinting of cancer systems continues to advance, a cancer system bioprinting platform is expected to become feasible once the challenging factors are overcome. Accordingly, bioprinting technology is expected to offer novel and better regulated cancer systems for increasing the throughput of drug discovery platforms and personalized drug tests.
